# Supplementary material for: Multi-scale dynamic imaging reveals that cooperative motility behaviors promote efficient predation in bacteria
Source: Nat Commun. 2023 Sep 11;14:5588. doi: 10.1038/s41467-023-41193-x (PMC10495355; doi:10.1038/s41467-023-41193-x)
Supplement: Supplementary file 1 — Supplementary Information [file 41467_2023_41193_MOESM1_ESM.pdf]

Supplementary Information for

Multi-scale dynamic imaging reveals that cooperative motility behaviors promote efficient predation in bacteria

Sara Rombouts[1], Anna Mas[1], Antoine Le Gall[1], Jean-Bernard Fiche[1], Tâm Mignot[2], Marcelo Nollmann[1]

[1] *Centre de Biochimie Structurale, CNRS UMR 5048, INSERM U1054, Université de Montpellier, 60 rue de Navacelles, 34090, Montpellier, France*
[2] *Laboratoire de Chimie Bactérienne, Marseille, France*

Table of contents

Supplementary Figure 1	**2-6**
Supplementary Figure 2	**7**
Supplementary Figure 3	**8**
Supplementary Figure 4	**9-10**
Supplementary Figure 5	**11**
Supplementary Table 1	**12**

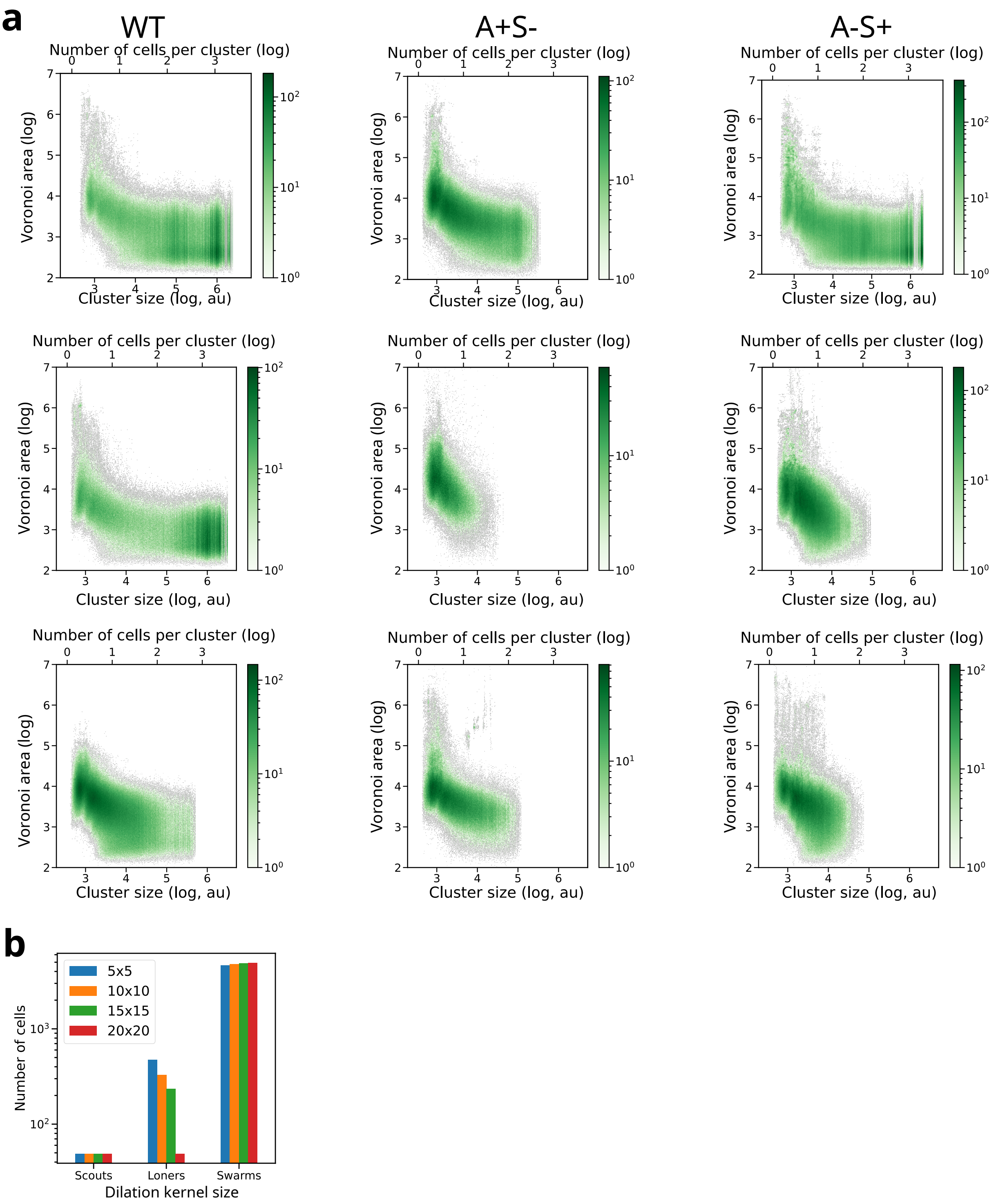

Supplementary figure 1 :

a) Examples of 2D Voronoi area-cluster size histograms for wild-type, S-motile cells (A-S+) and A-motile (A+S-) cells from experimental replicates (left, middle and right columns, respectively).

b) Effect of multiscale clustering dilation Kernel size parameter on the classification of cells. Histograms show the number of cells in either scout, loner or swarm classes for four distrinct dilation kernel size values.

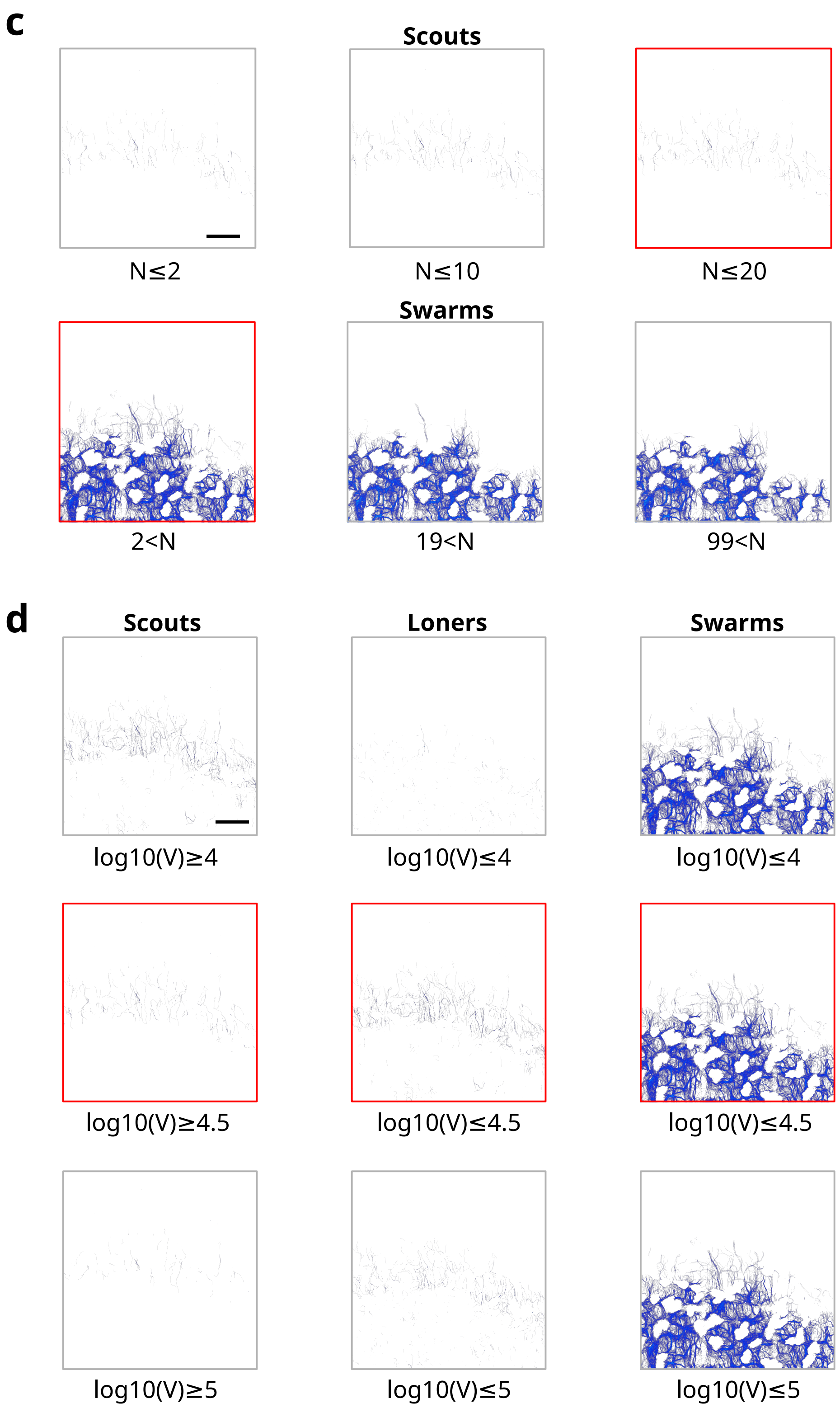

Supplementary figure 1 :

c) Spatial occupation of scout (top row) and swarm (bottom row) trajectories for wildtype conditions at the predation forefront for distincts thresholds values of cells per cluster (N). Scalebars = 100 µm. The red boxes represent the conditions used for the analyses in this article.

d) Spatial occupation of scout (top row), loner (middle row) and swarm (bottom row) trajectories for wildtype conditions at the predation forefront for distinct threshold values of voronoi areas (log10(V)). Scalebars = 100 µm. The red boxes represent the conditions used for the analyses in this article.

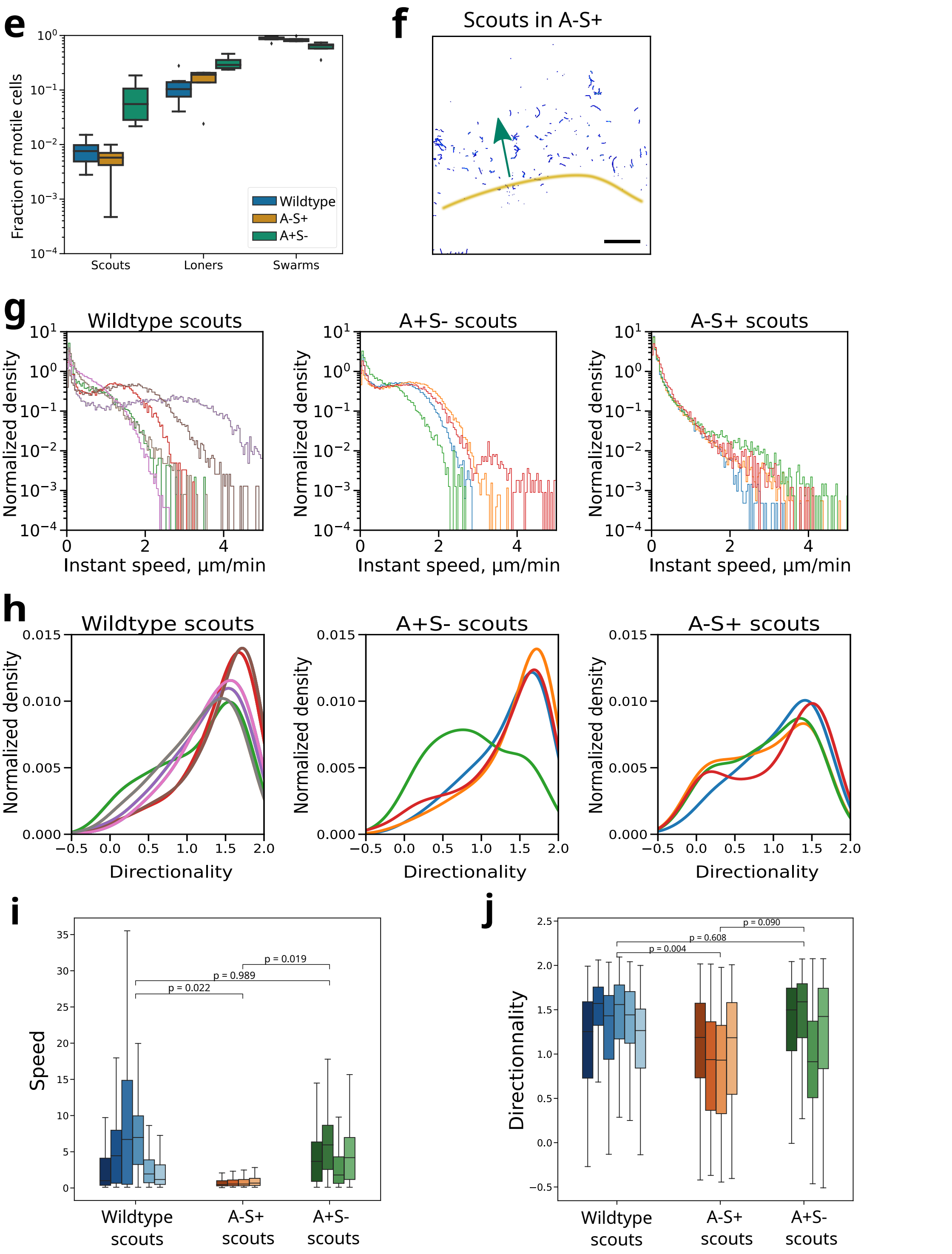

Supplementary figure 1 :

e) Box plot summaries of the fraction of motile cells for each class in wildtype (WT, blue boxes), S-motile (A-S+, orange boxes) and A-motile (A+S-, green boxes) communities. Each boxplot represents the statistics across six experimental replicates for the wildtype and four replicates for each mutant strains. Boxes show the median and the interquartile range, while whiskers represent the minimum and maximum values in the datasets. The corresponding statistical tests calculated using a two-sided t-test are p=0.397 between WT and A-S+ scouts, p=0.043 between WT and A+S- scouts; p= 0.608 between WT and A-S+ loners, p=0.011 between WT and A+S- loners; p=0.647 between WT and A-S+ swarms, p=0.011 between WT and A+S- swarms.

f) Histogram of gyration radii of scouts cells trajectories in wildtype, A-motile (A+S-) and S-motile (A-S+) communities. Shaded areas highlight the standard deviations from the mean (solid line) of six experimental replicates for the wild-type and four replicates for each mutant strains.

g) Histograms of instantaneous speed of scout cells from experimental replicates used in the main figure for wild-type, S-motile cells (A-S+) and A-motile (A+S-) cells. While isolated and small groups of S-motile cells can be found ahead of the predation front, A-motility is required for their mobility in regions avoid of prey.

h) Histograms of directionality of scout cells from experimental replicates used in the main figure for wild-type, S-motile cells (A-S+) and A-motile (A+S-) cells.

i) Box plot summaries of the speed of scout cells in wildtype (blue boxes), S-motile (A-S+, orange boxes) and A-motile (A+S-, green boxes) communities. Each boxplot represents the statistics across six experimental replicates for the wildtype and four replicates for each mutant strains. Boxes show the median and the interquartile range, while whiskers represent the minimum and maximum values in the datasets. The statistics were calculated using a two-sided t-test.

j) Box plot summaries of the directionality of and scout cells in wildtype (blue boxes), S-motile (A-S+, orange boxes) and A-motile (A+S-, green boxes) communities. Each boxplot represents the statistics across six experimental replicates for the wildtype and four replicates for each mutant strains. Boxes show the median and the interquartile range, while whiskers represent the minimum and maximum values in the datasets. The statistics were calculated using a two-sided t-test.

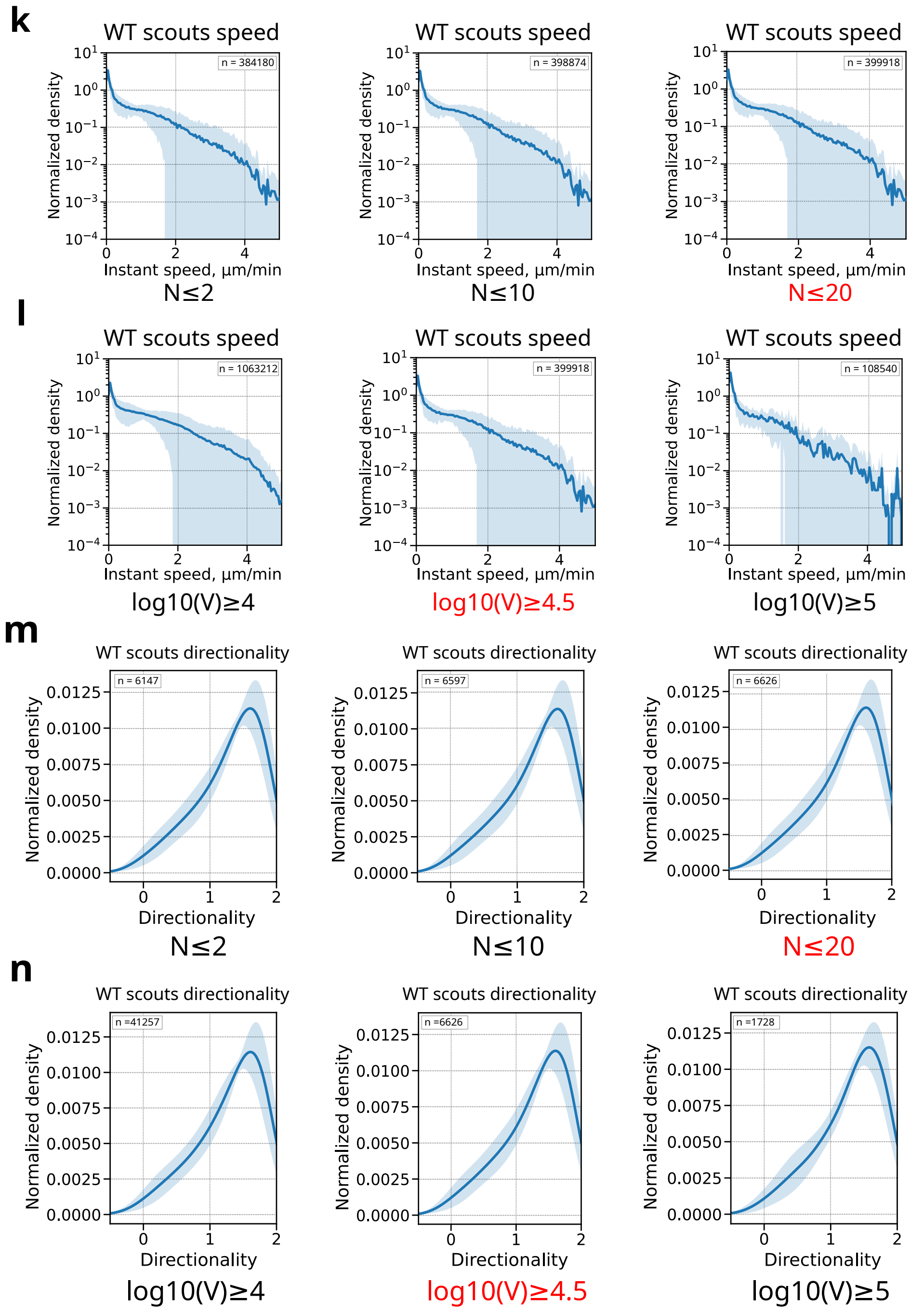

Supplementary figure 1 :

k) Histogram of instantaneous speed of scouts cells in wildtype conditions for distinct threshold values of cells per cluster (N) . Shaded areas highlight the standard deviations from the mean (solid line) of six experimental replicates. n represents the number of cells. The red writings represent the conditions used for the analyses in this article.

l) Histogram of instantaneous speed of scouts cells in wildtype conditions for distinct threshold values of voronoi areas (log10(V)). Shaded areas highlight the standard deviations from the mean (solid line) of six experimental replicates. n represents the number of cells. The red writings represent the conditions used for the analyses in this article.

m) Histograms of directionality of scout cells in wildtype conditions for distinct threshold values of cells per cluster (N). Shaded areas highlight the standard deviations from the mean (solid line) of six experimental replicates. n represents the number of trajectories. The red writings represent the conditions used for the analyses in this article.

n) Histogram of directionality of scouts cells in wildtype conditions for distinct threshold values of voronoi areas (log10(V)). Shaded areas highlight the standard deviations from the mean (solid line) of six experimental replicates. n represents the number of trajectories. The red writings represent the conditions used for the analyses in this article.

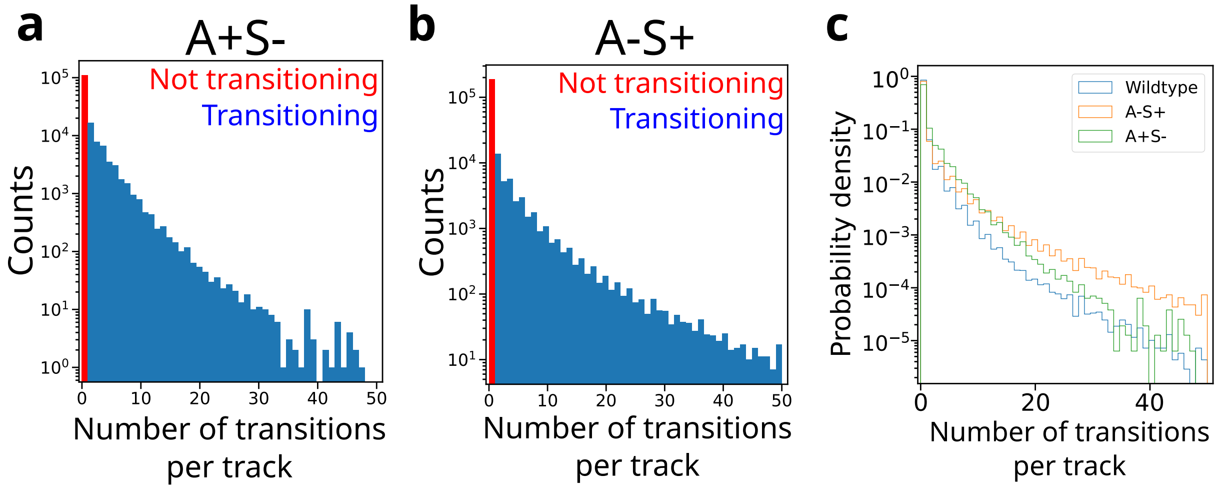

Supplementary figure 2 :

a) Histogram of number of motile cells transitions per trajectory for A-motile cells (A+S-) data accumulated from 4 experimental replicates.

b) Histogram of number of motile cells transitions per trajectory for S-motile cells (A-S+) data accumulated from 4 experimental replicates.

c) Normalized histograms of the probability of the number of transitions per trajectory for wild-type, S-motile cells (A-S+) and A-motile (A+S-) cells data accumulated from six experimental replicates for the wildtype and four replicates for each mutant strains.

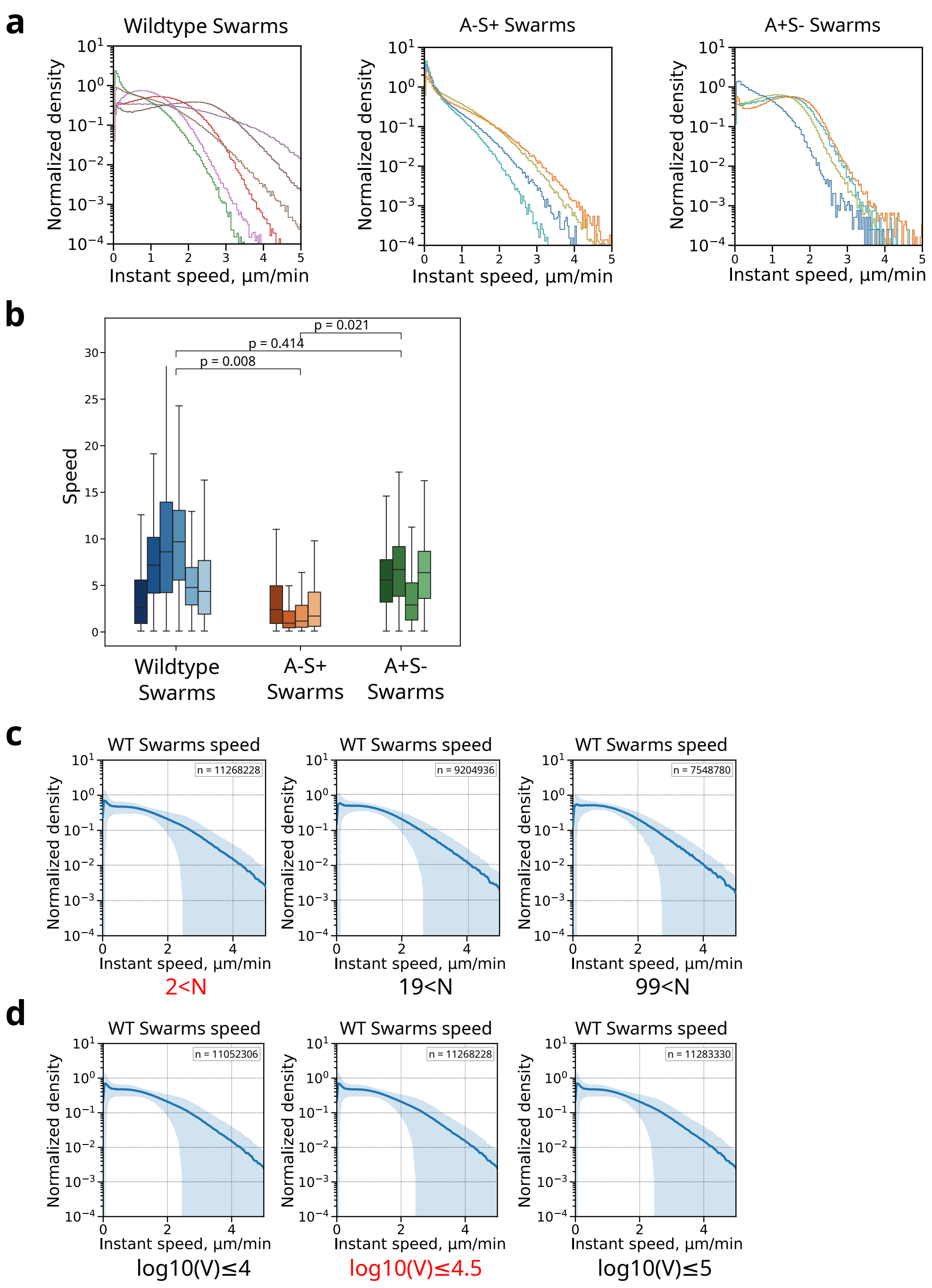

Supplementary figure 3 :

a) Histograms of instantaneous speed of swarm cells from experimental replicates used in the main figure for wild-type, S-motile cells (A-S+) and A-motile (A+S-) cells.

b) Box plot summaries of the speed of swarm cells in wildtype (blue boxes), S-motile (A-S+, orange boxes) and A-motile (A+S-, green boxes) communities. Each boxplot represents the statistics across six experimental replicates for the wildtype and four replicates for each mutant strains. Boxes show the median and the interquartile range, while whiskers represent the minimum and maximum values in the datasets. The statistics were calculated using a two-sided t-test.

c) Histogram of instantaneous speed of swarms cells in wildtype conditions for distinct threshold values of cells per cluster (N) . Shaded areas highlight the standard deviations from the mean (solid line) of six experimental replicates. n represents the number of cells. The red writings represent the conditions used for the analyses in this article.

d) Histogram of instantaneous speed of swarms cells in wildtype conditions for distinct threshold values of voronoi areas (log10(V)). Shaded areas highlight the standard deviations from the mean (solid line) of six experimental replicates. n represents the number of cells. The red writings represent the conditions used for the analyses in this article.

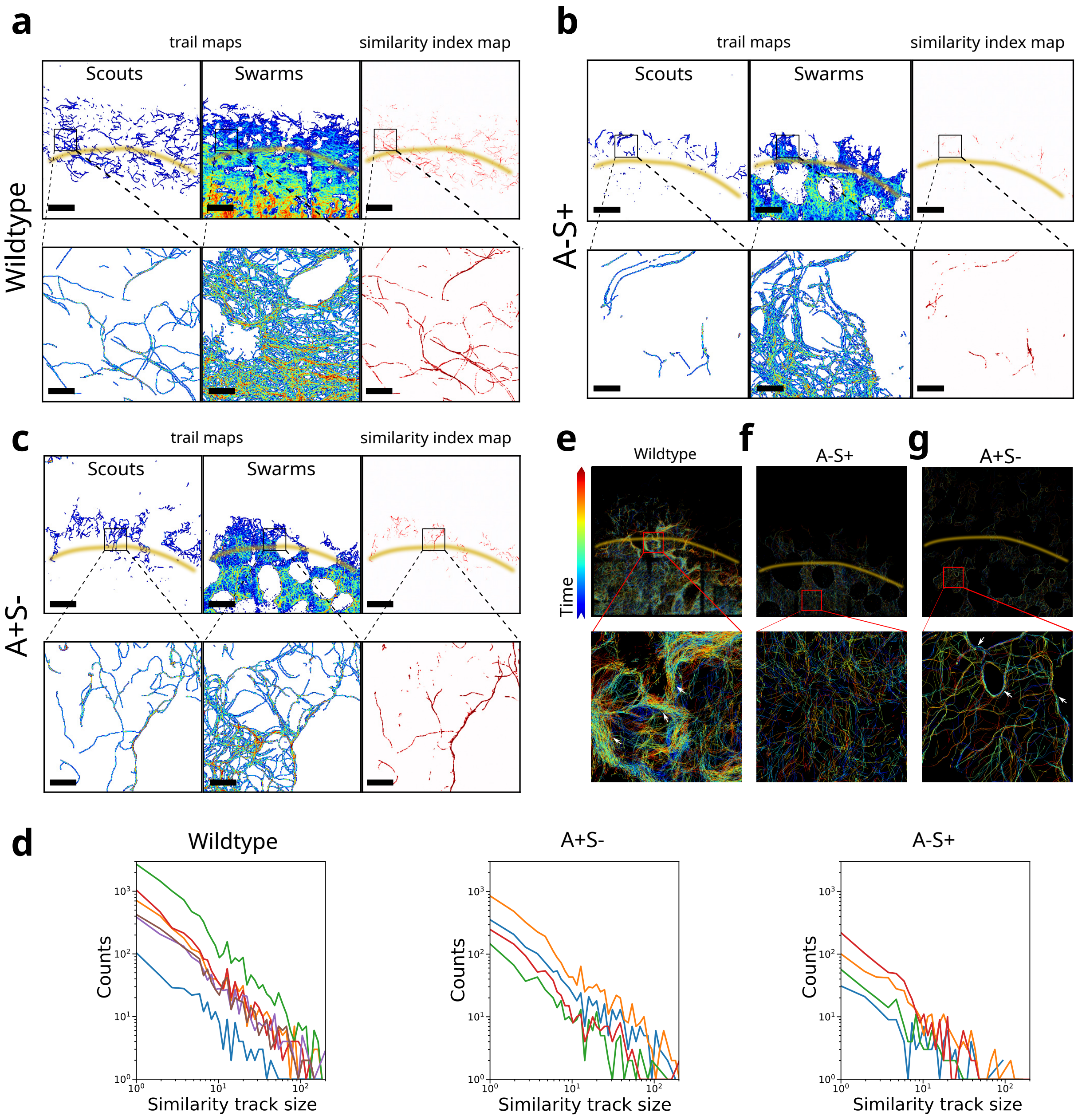

Supplementary figure 4 :

a-c) Examples of trail maps of scout and swarm cells and similarity index map for wildtype (A), A-S+ (B) and A+S- cells (C). Yellow lines delimitate the predation front. Scalebars = 100 µm. Scalebars of zoomed boxed areas = 20 µm

d) Histogram of the length of overlapping tracks (Similarity track size) used in the main figure for wild-type, S-motile cells (A-S+) and A-motile (A+S-) cells.

e-g) Examples of overlays of all trajectories for wildtype (D), S-motile (A-S+) cells (E) and A-motile (A+S-) cells (F). Yellow lines delimitate the predation front. White arrows in the zoom of the boxed areas point to examples of trails.

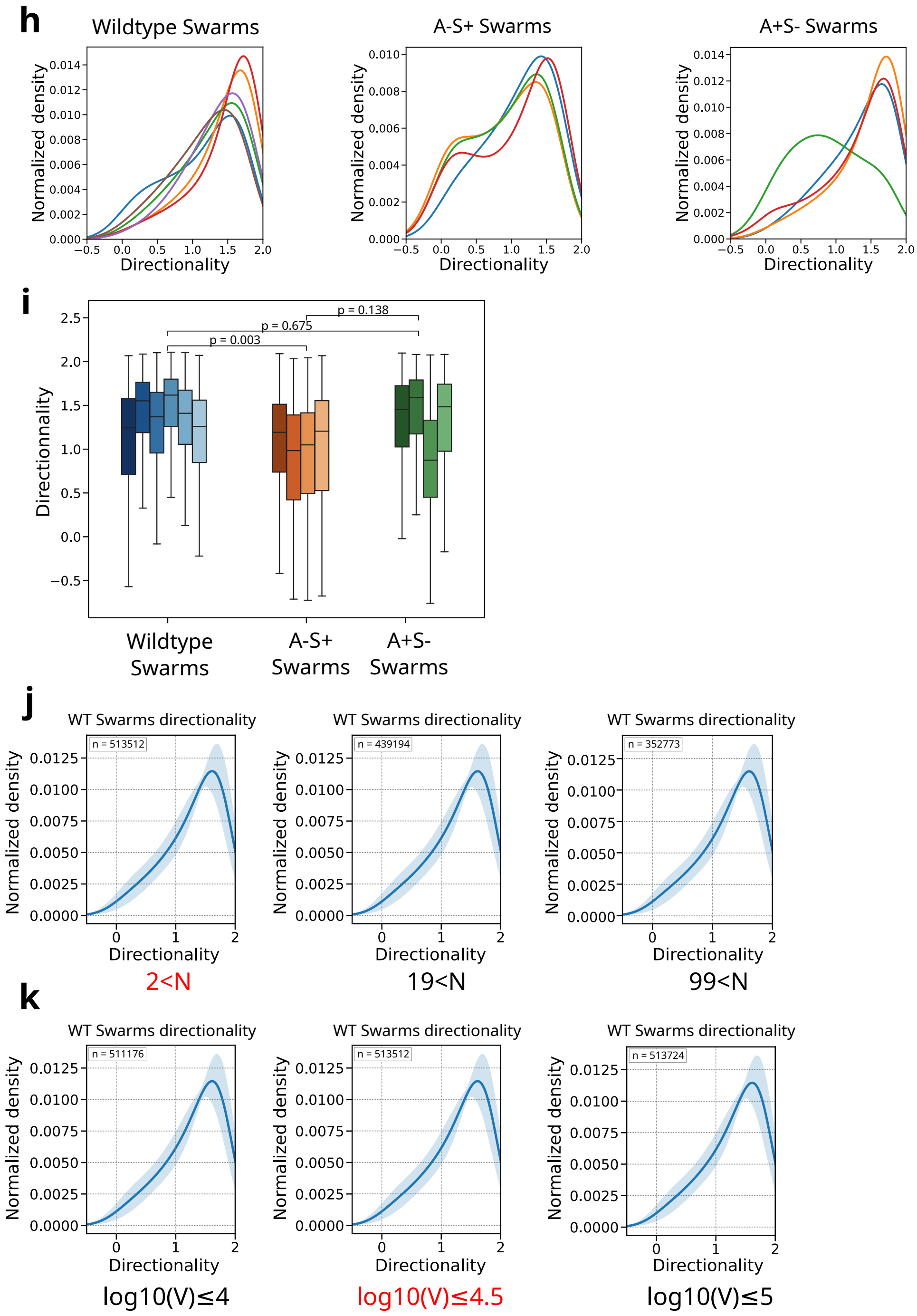

Supplementary figure 4 :

h) Histogram of swarm cells tracks directionality used in the main figure for wild-type, S-motile cells (A-S+) and A-motile (A+S-) cells.

i) Box plot summaries of the directionality of swarm cells in wildtype (blue boxes), S-motile (A-S+, orange boxes) and A-motile (A+S-, green boxes) communities. Each boxplot represents the statistics across six experimental replicates for the wildtype and four replicates for each mutant strains. Boxes show the median and the interquartile range, while whiskers represent the minimum and maximum values in the datasets. The statistics were calculated using a two-sided t-test.

j) Histograms of directionality of swarms cells in wildtype conditions for distinct threshold values of cells per cluster (N). Shaded areas highlight the standard deviations from the mean (solid line) of six experimental replicates. n represents the number of trajectories. The red writings represent the conditions used for the analyses in this article.

k) Histogram of directionality of swarms cells in wildtype conditions for distinct threshold values of voronoi areas (log10(V)). Shaded areas highlight the standard deviations from the mean (solid line) of six experimental replicates. n represents the number of trajectories. The red writings represent the conditions used for the analyses in this article.

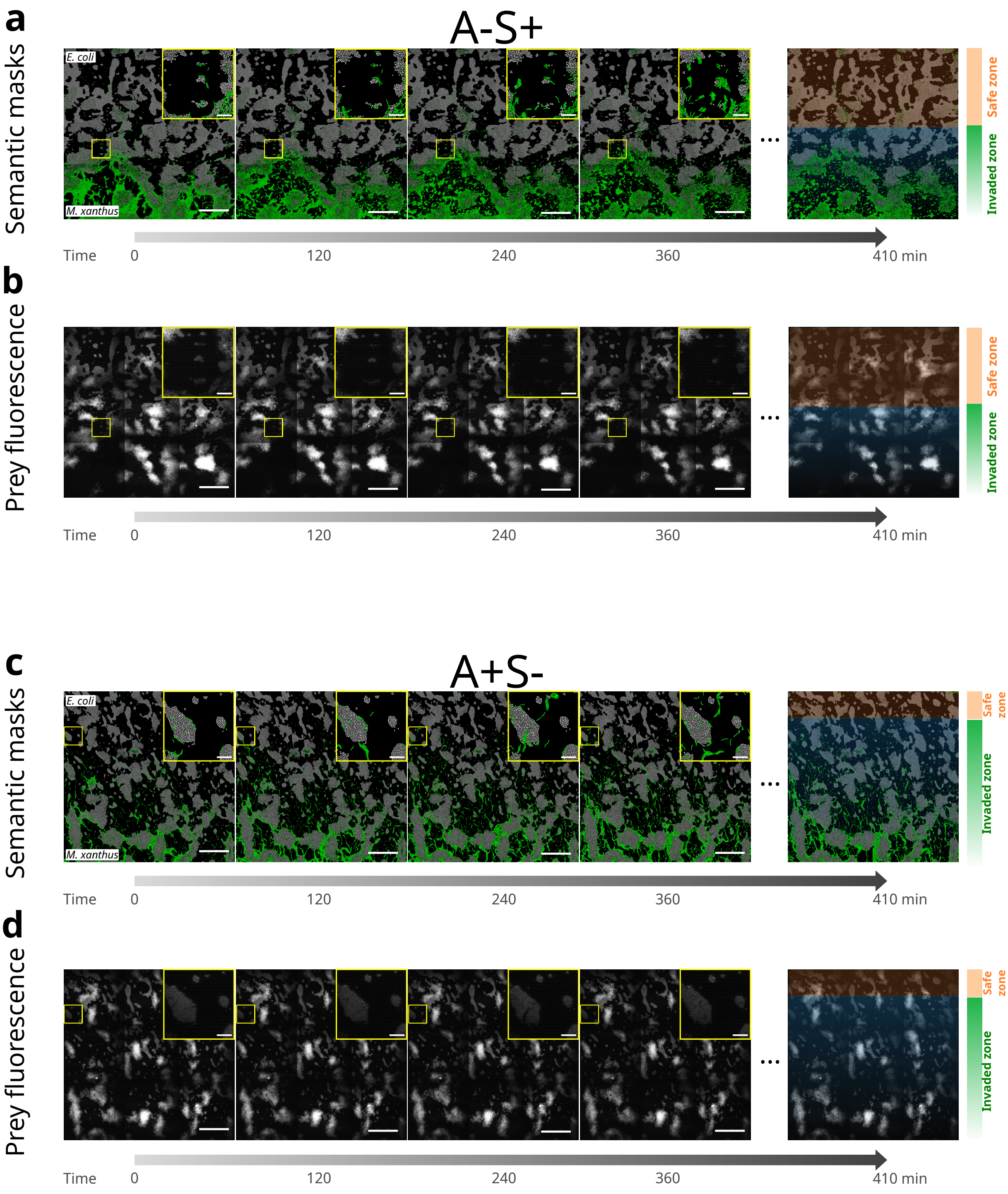

Supplementary figure 5 :

a) Evolution of predation over time at the predation forefront of S-motile cells (A-S+) visualized with semantically segmented large ROI containing the masks for *M. xanthus* (green) and *E. coli* (white). Two zones were defined: i) where no invasion occurred (orange shaded area) and ii) where *M. xanthus* cells invaded *E. coli* (blue shaded area). Scalebars = 100 µm. Scalebars of zoomed images = 20 µm

b) Evolution of the total raw fluorescence signal from *E. coli* cells over time invaded by S-motile *M. xanthus* cells (A-S+). Orange and blue boxed areas highlight the safe zone, where no predation occurred and the predation zone, where active predation is occurring, respectively. Scalebars = 100 µm. Scalebars of zoomed images = 20 µm

c-d) Same as panels A and B but for A-motile *M. xanthus* cells (A+S-)

Strain	Genotype	Strain origin
E. coli	MG1655 wildtype	Espeli laboratory collection
E. coli HU-mCherry	MG1655 HU-mCherry	Espeli laboratory collection
M. xanthus	DZ2 wildtype	Mignot laboratory collection
M. xanthus cytosolic-sfGFP	DZ2 pSWU19-Pm1000-sfGFP	Mignot laboratory collection
M. xanthus A+S- OMss-sfGFP	DZ2 ΩpilA pSWU19-PpilA-OMss-sfGFP	Mignot laboratory collection
M. xanthus A-S+ OMss-sfGFP	DZ2 GltJ DNterm222 pSWU19-PpilA-OMss-sfGFP	Mignot laboratory collection
M. xanthus A+S- OMss-mCherry	DZ2 DpilA pSWU19-PpilA-OMss-mCherry	Mignot laboratory collection
M. xanthus AglZ-NeonGreen	Allelic replacement of *aglZ* by *aglZ-NeonGreen*	Mignot laboratory collection

Supplementary Table 1 :

Bacterial strains used in this study
